# Independent factors associated with wearing different types of outdoor footwear in a representative inpatient population: a cross-sectional study

**DOI:** 10.1186/s13047-018-0260-7

**Published:** 2018-05-29

**Authors:** Alex L. Barwick, Jaap J. van Netten, Lloyd F. Reed, Peter A. Lazzarini

**Affiliations:** 1 School of Health and Human Sciences, Southern Cross University, Southern Cross Drive, Bilinga, QLD 4225 Australia; 2 School of Clinical Sciences, Queensland University of Technology, Brisbane, QLD Australia; 3 Institute of Health and Biomedical Innovation, Queensland University of Technology, Brisbane, QLD Australia; 4 Department of Rehabilitation, Academic Medical Center, University of Amsterdam, Amsterdam Movement Sciences, Amsterdam, the Netherlands; 5 Wound Management Innovation Cooperative Research Centre, Brisbane, QLD Australia; 6 Allied Health Research Collaborative, Metro North Hospital and Health Service, Brisbane, QLD Australia

**Keywords:** Footwear, Inpatient, Sex, Arthritis, Neuropathy, Diabetes, Running shoes, Flip flops, Walking shoes, Sandals

## Abstract

**Background:**

Footwear can have both a positive and negative impact on lower limb health and mobility across the lifespan, influencing the risk of foot pain, ulceration, and falls in those at risk. Choice of footwear can be influenced by disease as well as sociocultural factors, yet few studies have investigated the types of footwear people wear and the profiles of those who wear them. The aim of this study was to investigate the prevalence and factors associated with outdoor footwear type worn most often in a representative inpatient population.

**Methods:**

This study was a secondary data analysis of a cohort of 733 inpatients that is highly representative of developed nations’ hospitalised populations; 62 ± 19 years, 55.8% male, and 23.5% diabetes. Socio-demographic, medical history, peripheral arterial disease, peripheral neuropathy, foot deformity, foot ulcer history, amputation history and past foot treatment variables were collected. Participants selected the footwear type they mostly wore outside the house in the previous year from 16 types of footwear. Multivariate logistic regression identified independent factors associated with outdoor footwear types selected.

**Results:**

The most common outdoor footwear types were: running shoes (20%), thongs/flip flops (14%), walking shoes (14%), sandals (13%) and boots (11%). Several socio-demographic, medical history and foot-related factors were independently associated (Odds Ratio; 95% Confidence Interval)) with different types of footwear. Running shoes were associated with male sex (2.7; 1.8–4.1); thongs with younger age (0.95 for each year; 0.94–0.97), being female (2.0; 1.2–3.1) and socio-economic status (3.1; 1.2–7.6); walking shoes with arthritis (1.9; 1.2–3.0); sandals with female sex (3.8; 2.3–6.2); boots with male sex (9.7; 4.3–21.6) and inner regional (2.6; 1.3–5.1) and remote (3.4; 1.2–9.5) residence (all, *p* < 0.05).

**Conclusions:**

We profiled the types of outdoor footwear worn most in a large diverse inpatient population and the factors associated with wearing them. Sex was the most consistent factor associated with outdoor footwear type. Females were more likely to wear thongs and sandals and males boots and running shoes. Overall, this data gives insights into the socio-demographic, medical and other health factors that are related to footwear choice in a large diverse population primarily of older age.

**Electronic supplementary material:**

The online version of this article (10.1186/s13047-018-0260-7) contains supplementary material, which is available to authorized users.

## Background

Footwear can impact lower limb health and general mobility both positively and negatively across the lifespan [1]. Different footwear features have an effect on the biomechanics of standing and gait and hence can influence musculoskeletal function and dysfunction [2, 3]. As a result, footwear is of relevance to a diverse range of population groups. Certain footwear can contribute to the development of pain [4], complications of diabetes including ulceration [5], and imbalance that increases the risk of falls [6, 7]. Accordingly, footwear displaying certain features are often recommended in the prevention and management of these conditions in specific populations [8–12].

Footwear can be classified based on distinctive combinations of features into types such as sandals and boots [13]. Outdoor footwear requires features that protect the foot from the external environment, but has further requirements to promote lower limb health and mobility. Such requirements include: adequate width, depth and length to accommodate the foot; a soft, flexible and protective upper; low heel height; stable heel counter and limited available torsion for overall shoe stability; adequate outsole grip to prevent slipping; and being fit for purpose [1, 12]. Footwear also has individualised psychosocial requirements, as choice of footwear type is also influenced by sociocultural, psychological and other health factors [14, 15].

For some populations complying with recommended footwear features can be challenging, such as older people and those with arthritis. For example, foot deformity may change the shape of the foot causing difficulty in fitting standard prefabricated footwear [16]. Such constraints in footwear choices have also been shown to affect individuality, well-being and quality of life [16].

Some previous research has investigated the outdoor footwear worn by specific patient groups. Those with arthritis have been found primarily to wear athletic or walking shoes [17, 18] and sandals [17, 19]. However, many people with arthritis [19, 20], diabetes [21, 22], and older people [23] often also wear inadequate footwear including thongs/flip flops [19–22] and slippers [22], or even go barefoot [21, 22]. To our knowledge, no previous research has investigated the outdoor footwear worn in a large diverse population and the profiles of those who wear them. Information on the types of people who wear certain footwear in a representative inpatient population may provide a starting point for further research into potential causal influences on footwear choices that could be used to guide footwear behaviour change interventions in future.

## Methods

The aim of this study was to investigate the prevalence and factors independently associated with different outdoor footwear types worn most in the year prior to hospitalisation in a large representative inpatient population. This was a secondary data analysis of a multi-site cross-sectional observational study that investigated foot disease in an inpatient population, and has been described in detail elsewhere [24, 25]. Briefly, on one designated study day, all adult inpatients admitted into hospital for any medical reason (except those with cognitive deficits, in maternity and in psychiatric wards) in five public hospitals in Queensland (Australia) were invited to participate [24]. 883 eligible participants were invited and 733 (83%) consented. The demographic, social determinant and medical history make-up of this sample has been reported to be highly representative of typical inpatient populations present in developed nations [24–26]. Self-reported history and foot physical examination was performed using a validated data collection instrument (the Queensland Foot Disease Form) [24, 25, 27]. The items contained in this instrument have demonstrated at least moderate criterion validity, inter and intra-rater reliability in two different studies [24, 27].

The self-reported explanatory variables were grouped into the domains of socio-demographics (age, sex, indigenous status, country of birth, socioeconomic status, geographical remoteness), medical conditions history (diabetes, hypertension, dyslipidaemia, myocardial infarct, cerebrovascular accident, chronic kidney disease, cancer, arthritis, depression, smoking, mobility impairment, vision impairment), and past foot treatment in the year prior to hospitalisation (by podiatrist, general practitioner, specialist physician, surgeon, nurse, orthotist and other) [24, 25].

The clinically-diagnosed explanatory variables were all foot-related conditions and obtained following physical examination, including: amputation history, foot ulcer history (current or previous), peripheral artery disease (PAD) severity, peripheral neuropathy and foot deformity. PAD severity was diagnosed based on a toe systolic pressure of < 70 mmHg, as mild (51-70 mmHg), moderate (31-50 mmHg) and critical (< 30 mmHg) PAD [28, 29]. Peripheral neuropathy was diagnosed as the failure to sense a 10-g monofilament on at least two or more plantar forefoot sites on one foot [30, 31]. The presence of three or more of the following in one foot was the basis for the diagnosis of a foot deformity: small muscle wastage, bony prominence, prominent metatarsal heads, hammer or claw toes, limited joint mobility or Charcot deformity on one foot [30, 32].

The outcome variable for this study was the self-reported footwear type worn most outside in the previous 12 months. Each participant was presented with a validated footwear type picture chart [13], modified with permission to add drawings of socks only and barefoot (no footwear) options. Participants were asked “from this chart displaying 16 different types of footwear, what is the type of shoes you have worn most outside the house over the past 12 months?” [24, 25]. The chart displayed drawings and titles of walking shoes, running shoes, oxford shoes, moccasins, boots, ugg boots, high heels, thongs/flip flops, slippers, backless slippers, court shoes, mules, sandals, bespoke footwear, socks only, and barefoot [13]. Participants were asked to select one type of footwear only [24, 25].

### Statistical analysis

All data were analysed using SPSS 22.0 for Windows (SPSS Inc., Chicago, IL, USA) or GraphPad Prism (GraphPad Software Inc., San Diego, CA, USA). Descriptive statistics were used to display all variables. Prevalence with 95% Confidence Intervals (95% CI) was evaluated for all footwear outcome variables. Associations between explanatory and outcome variables were analysed using univariate logistic regression. All variables achieving a statistical significance of *p* < 0.2 were included in backwards stepwise multivariate logistic regression analysis until only variables reaching statistical significance remained (*p* < 0.05) (Unadjusted Model) [24, 33, 34]. The unadjusted model was then adjusted for age, sex, socio-economic status and geographical remoteness by entering these variables into the model with the variables remaining in the unadjusted model (Adjusted Model) [24, 33, 34]. Collinearity, goodness of fit, significance, parsimony and variance were assessed at each step and found to be acceptable [33, 34]. Cases with missing data were excluded, as the proportion of missing data cases was minimal (< 5% in all cases) [24, 33, 34].

## Results

Table 1 displays the numbers and prevalence (% and 95 CI) of each of the 16 different types of outdoor footwear. Participant characteristics and univariate analyses for each footwear type with a prevalence of > 1% are presented in Supplementary Tables S1-S4 (Additional file 1). Table 2 displays the results of the multivariate logistic regression of unadjusted and adjusted models for each footwear type. Outdoor footwear types with ≤1% prevalence (backless slipper (1%; 0–2.0), ugg boots (0.8%; 0–1.8), socks only (0.4%; 0–1.3), mules (0.3%; < 0–1.1) and high heels (0.1%; < 0–0.9) were not entered into univariate or multivariate analyses.

**Table 1:** Main outdoor footwear types worn in the previous 12 months

Rank	Footwear Type	Number	% (95% CI)
1	Running shoe	148	20.4% (17.6–23.5)
2	Thongs/flip flops	103	14.2% (11.8–16.9)
3	Walking shoe	98	13.5% (11.2–16.2)
4	Sandal	95	13.1% (10.8–15.7)
5	Boot	78	10.7% (8.7–13.2)
6	Oxford shoe	50	6.9% (5.3–9.0)
7	Court shoe	49	6.7% (5.1–8.8)
8	Moccasin	42	5.8% (4.2–7.7)
9	Slipper	20	2.8% (1.8–4.2)
10	Bespoke footwear	12	1.7% (0.9–2.9)
11	Barefoot	12	1.7% (0.9–2.9)
12	Backless slipper	7	1.0% (0–2.0)
13	Ugg boot	6	0.8% (0–1.8)
14	Socks only	3	0.4% (0–1.3)
15	Mule	2	0.3% (< 0–1.1)
16	High heel	1	0.1% (< 0–0.9)
Total		726	100%

**Table 2:** Independent factors associated with outdoor footwear type worn most in the past 12 months (Odds Ratios [95% CI])

Risk Factor	Unadjusted	*p* Value	Adjusted	*p* Value
Running Shoes				
Male	2.65 [1.77–3.95]	< 0.001	2.69 [1.79–4.05]	< 0.001
Thongs/flip flops				
Age (year)	0.96 [0.94–0.97]	< 0.001	0.95 [0.94–0.97]	< 0.001
Female	1.81 [1.15–2.85]	0.011	1.95 [1.23–3.11]	0.005
Socioeconomic status		0.010		0.055
Least disadvantaged	1.00		1.00	
Second least disadvantaged	3.22 [1.41–7.33]	0.005	3.05 [1.23–7.56]	0.016
Middle	2.14 [0.98–4.69]	0.057	2.05 [0.90–4.67]	0.086
Second most disadvantaged	2.14 [0.90–5.09]	0.084	1.72 [0.68–4.33]	0.252
Most disadvantaged	1.11 [0.51–2.43]	0.791	1.08 [0.48–2.39]	0.857
Walking shoes				
Arthritis	2.23 [1.45–3.43]	0.001	1.92 [1.21–3.03]	0.005
Sandals				
Female	3.52 [2.18–5.67]	< 0.001	3.78 [2.30–6.22]	< 0.001
Non-Smoker	15.94 [2.19–116.19]	0.006	Overfitted	
Boots				
Male	9.35 [4.21–20.73]	< 0.001	9.67 [4.33–21.64]	< 0.001
Geographic Remoteness		0.008		0.031
Major city	1.00		1.00	
Inner regional area	2.47 [1.37–4.44]	0.003	2.57 [1.29–5.13]	0.007
Outer regional area	2.05 [0.93–4.52]	0.074	2.16 [0.92–5.09]	0.078
Remote area	3.05 [1.18–7.91]	0.022	3.38 [1.20–9.53]	0.022
Very remote area	3.04 [1.02–9.06]	0.047	2.84 [0.82–9.89]	0.101
Oxford Shoes				
Age	1.02 [1.00–1.04]	0.025	1.02 [1.01–1.04]	0.013
Male	6.62 [2.78–15.78]	< 0.001	6.73 [2.79–16.20]	< 0.001
Court shoes^a^				
Age	1.04 [1.02–1.06]	0.001	1.03 [1.01–1.05]	0.008
No Smoking History	2.20 [1.13–4.28]	0.020	2.06 [1.04–4.08]	0.039
Past Podiatry Treatment	2.22 [1.18–4.19]	0.014	2.58 [1.32–5.02]	0.005
Moccasins				
Female	2.13 [1.12–4.05]	0.021	2.00 [1.05–3.83]	0.036
Slippers				
Male	6.66 [1.52–29.09]	0.012	7.31 [1.65–32.38]	0.009
Chronic kidney disease	4.50 [1.77–11.47]	0.002	3.34 [1.22–9.10]	0.019
Bespoke shoes				
Past podiatry treatment	9.68 [2.59–36.14]	0.001	13.86 [3.03–63.46]	0.001
Barefoot				
Age	0.96 [0.93–0.99]	0.018	0.95 [0.01–0.99]	0.010
Peripheral neuropathy	8.84 [2.44–32.09]	0.001	7.51 [1.60–35.22]	0.011

^a^Sex removed from model as all but one person wearing court shoes were female; Missing: Excluded missing cases

### Running shoes

Running shoes were worn by 20.4% (17.6–23.5) of participants. Running shoes had univariate associations with: male sex, depression and past podiatry treatment (all, *p* < 0.02) (Supplementary Table S1). In the adjusted multivariate model (OR; 95% CI), running shoes were independently associated with being male (2.7; 1.8–4.1; *p* < 0.001).

### Thongs/flip flops

Thongs/flip flops were worn by 14.2% (11.8–16.9) of participants. Thongs had univariate associations with: age, female sex, second least disadvantaged socioeconomic status, outer regional residence, arthritis, depression, smoking, mobility impairment, past foot treatment by a podiatrist, peripheral neuropathy, foot deformity and mild and moderate PAD (all, *p* < 0.05) (Supplementary Table S1). In the adjusted multivariate model, thongs were independently associated with younger age (0.95 per year; 0.94–0.97), female sex (2.0; 1.2–3.1) and the second least disadvantaged socioeconomic group (3.1; 1.2–7.6; all, *p* < 0.05).

### Walking shoes

Walking shoes were worn by 13.5% (11.2–16.2) of the participants. Walking shoes had univariate associations with: age, cerebrovascular accident, arthritis, mobility impairment, past foot treatment by a podiatrist, and foot deformity (all, *p* < 0.05) (Supplementary Table S1). In the adjusted multivariate model, walking shoes were independently associated with arthritis (1.9; 1.2–3.0; *p* = 0.005).

### Sandals

Sandals were worn by 13.1% (10.8–15.7) of the participants. Sandals had univariate associations with: age, female sex, smoking and mobility impairment (all, *p* < 0.05) (Supplementary Table S2). In the adjusted multivariate model, sandals were independently associated with female sex (3.8; 2.3–6.2; *p* < 0.01).

### Boots

Boots were worn by 10.7% (8.7–13.2) of participants. Boots had univariate associations with: age, male sex, inner regional, outer regional, remote and very remote residence, smoking, mobility impairment, past foot treatment by a podiatrist, peripheral neuropathy and foot deformity (all, *p <* 0.05) (Supplementary Table S2). In the adjusted multivariate model, boots were independently associated with male sex (9.7; 4.3–21.6; *p* < 0.001), inner regional residence (2.6; 1.3–5.1, *p* = 0.007) and remote area residence (3.4; 1.2–9.5; *p* = 0.022).

### Oxford shoes

Oxford shoes were worn by 6.9% (5.3–9) of the participants. Oxford shoes had univariate associations with: age, male sex and being born overseas (all, *p* < 0.05) (Supplementary Table S2). In the adjusted multivariate model, oxford shoes were independently associated with older age (1.02 per year; 1.01–1.04; *p* = 0.013) and male sex (6.7; 2.8–16.2; *p* < 0.001).

### Court shoes

Court shoes were worn by 6.7% (5.1–8.8) of the participants. Court shoes had univariate associations with: age, female sex, arthritis, history of smoking, mobility impairment and past foot treatment by a podiatrist (all, *p* < 0.05) (Supplementary Table S3). Sex was excluded from multivariate analyses as all but one participant that wore court shoes were female. In adjusted multivariate analyses, court shoes were independently associated with older age (1.03 per year; 1.01–1.05; *p* = 0.008), non-smoking history (2.1; 1.04–4.08; *p* = 0.039) and past podiatry treatment (2.6; 1.3–5.0; *p* = 0.005).

### Moccasins

Moccasins were worn by 5.8% (4.2–7.7) of participants. Moccasins had a univariate association with female sex (*p* = 0.021) (Supplementary Table S3). In the adjusted multivariate model, moccasins were independently associated with female sex (2.0; 1.1–3.8; *p* = 0.036).

### Slippers

Slippers were worn by 2.8% (1.8–4.2) of participants. Slippers had univariate associations with: age, male sex, chronic kidney disease and critical PAD (all *p <* 0.02) (Supplementary Table S3). In the adjusted multivariate model, slippers were independently associated with male sex (7.3; 1.7–32.4; *p* = 0.009) and chronic kidney disease (3.3; 1.2–9.1; *p* = 0.019).

### Bespoke shoes

Bespoke shoes were worn by 1.7% (0.9–2.9) of participants. Bespoke shoes had univariate associations with: diabetes, cerebrovascular accident, chronic kidney disease, past foot treatment including by a podiatrist, general practitioner, surgeon, physician, nurse or orthotist, amputation history, foot ulcer history, peripheral neuropathy, and foot deformity (all, *p* < 0.05) (Supplementary Table S4). In the adjusted multivariate model, bespoke shoes were independently associated with past podiatry treatment (13.9; 3.0–63.5; *p* = 0.001).

### No shoes (barefoot)

No shoes were worn by 1.7% (0.9–2.9) of participants. Wearing no shoes had a univariate association with peripheral neuropathy (*p* = 0.006) (Supplementary Table S4). In the adjusted multivariate model, wearing no shoes was independently associated with younger age (0.95 per year, 0.01–0.99; *p* = 0.01) and peripheral neuropathy (7.5; 1.6–35.2; *p* = 0.011).

## Discussion

Footwear is important to the maintenance of general mobility and lower limb health, with some footwear types more recommended than others in the treatment and prevention of foot-related disease [1, 17, 35]. This study sought to describe the outdoor footwear types worn most in the year prior to hospitalisation by a representative sample of adult inpatients, and to investigate the factors associated with their wear. The most commonly worn outdoor footwear type was running shoes which does fall within footwear recommendations for many pathological populations [24]. This was followed by thongs/flip flops, walking shoes, sandals and boots. Previous studies have also found running shoes, thongs/flip flops, walking shoes and sandals to be popular footwear amongst specific pathological populations including those with arthritis [17–19, 36], diabetes [22], and those at risk of falls [37]. Although comparison with these condition-specific studies is challenging due to the differing conditions, geographical locations and methods used to categorise footwear type, taken together it does appear that running shoes, thongs/flip flops, walking shoes and sandals are popular outdoor footwear in diverse populations.

We found that some socio-demographic factors, medical conditions, foot conditions and past foot treatment were independently associated with different outdoor footwear types worn. Male sex was independently associated with wearing running shoes (OR 2.7), boots (OR 9.7), oxford shoes (OR 6.7) and slippers (OR 7.3). Whereas, in stark contrast, female sex was associated with wearing thongs/flip flops (OR 2.0), sandals (OR 3.8), moccasins (OR 2.0) and court shoes (only one male participant wore court shoes). This is similar to previous research that has found marked differences in men’s and women’s preferred footwear [4, 38].

Although men and women have similar footwear needs, footwear type is chosen along gender lines, following sociocultural influences, rather than medical or foot conditions [14]. Oxford shoes and boots are traditionally male footwear, and sandals and court shoes traditionally female. While associations between different footwear types and gender are perhaps not surprising, our findings were from one of the first studies to adjust for multiple other factors (socio-demographic, medical, and foot condition factors). The gender relationships still remained in adjusted multivariate analyses. This may have relevant clinical consequences, with females much more likely to choose footwear types with features that are not in line with recommended characteristics for footwear that promotes general lower limb health and mobility. For example, sandals, court shoes and thongs/flip flops are less likely to have a protective upper, adequate outsole grip, stable heel counter and limited available torsion than running shoes, boots and oxford shoes [39]. Furthermore, women are more likely to report footwear difficulties [40] and pain when wearing footwear [41], with the types of footwear chosen likely a reason.

Age was also related to footwear choice, with both health and generational sociocultural factors likely to play a role in this relationship. Younger age was associated with increased likelihood of wearing thongs/flip flops (OR 0.95 per year of age) and going barefoot (OR 0.95 per year of age); while older age was associated with increased likelihood of wearing oxford shoes (OR 1.02 per year of age) and court shoes (OR 1.03 per year of age). A likely reason for this is that aesthetic footwear preferences are likely to be different in older generations compared to those in younger ones. Additionally, as people age they are more likely to value the health-promoting features of footwear such a comfort, stability and fit over aesthetics [17, 19].

There were several other associations observed among sociodemographic and outdoor footwear types. The observed association between wearing boots and living in a regional (OR 2.6) or remote (OR 3.4) area could be cultural and related to higher prevalence of occupations requiring the wearing of boots, such as farming and mining. The associations between thongs/flip flops and the second least disadvantaged socio-economic group (OR 3.1) and between court shoes and non-smoking (OR 2.6) have less clear potential explanations. Differing fashion trends across social groups might potentially be responsible. Future research should examine whether these relationships exist in other populations and include investigations of the motivations of these footwear choices.

Independent relationships were observed between past podiatry treatment and bespoke shoes (OR 13.9) and court shoes (OR 2.58). Someone requiring bespoke footwear is likely to have foot deformity and associated problems that necessitate treatment by a podiatrist. The reason for the relationship with court shoes is less clear. Unexpectedly, there was a large association (OR 7.5) between going barefoot and peripheral neuropathy in the 12 participants who indicated they primarily do not wear footwear outdoors. Further research should investigate whether this is a relationship that is present in the larger population as there are clinical implications. People who have lost protective sensation have significantly increased needs for footwear features that promote physical protection from external trauma and support to improve mobility [42].

Walking shoes were associated with arthritis (OR 2.2), similar to previous research that reported comfort and fit to be priorities when choosing footwear in this population [16, 17, 19]. Pain caused by arthritis may motivate the wearing of comfortable and stable walking shoes. Chronic kidney disease was associated with wearing slippers (OR 3.3); we hypothesise that this might be resulting from general ill-health and inability to don and doff shoes, or increased need to keep poorly perfused feet warm.

This study provides, for the first time, insights into the typical outdoor footwear worn in the year prior to being an inpatient and the factors associated with them. It does however, have several limitations. This was a secondary analysis of data from the *Foot Disease in Inpatients Study* [24, 25]. The large amount of analyses performed in this and previous papers using this large existing database does increase the risk of type 1 error. Inpatients are typically older and have more chronic conditions compared to the general population and our sample was highly representative of these characteristics. Thus, our findings are not as likely to be generalisable to unhospitalised populations; however, an older population is more vulnerable to foot-related conditions and thus a very relevant population to study in regards to footwear worn. Another limitation is that all sites were in Queensland, Australia, which has a tropical climate. The likely effect of this climate on a person’s year round footwear may influence the results, further limiting their generalisability. The cross-sectional nature of the study means causal pathways cannot be confirmed. Some common sense explanations have been discussed that further research should investigate these. The explanatory variables investigated reported high validity and reliability [24, 25]; however, although foot conditions were diagnosed using gold standard clinical testing, various others were self-reported. Lastly, the self-reported outcome of outdoor footwear type mostly worn in the previous year is vulnerable to recall bias and may not represent the range of outdoor footwear types worn by the participants’ as it only allowed for one footwear type to be selected.

## Conclusions

Running shoes, thongs/flip flops, walking shoes, sandals and boots were the most common outdoor footwear types prior to hospitalisation in a large representative inpatient population. Various socio-demographic, medical history and foot-related factors were identified as independently associated with outdoor footwear use in this study. Age and sex were most consistently linked with particular footwear types, with females and younger populations tending towards footwear that is not recommended for general mobility and lower limb health. Overall, our findings provide valuable new population-based insights into the socio-demographic and health factors that potentially influence people’s choice of outdoor footwear in a diverse population.

## Additional file

Additional file 1: **Tables S1-S4** include participant characteristics and univariate analyses for each footwear type with a prevalence of > 1% and are available. (DOCX 62 kb)

## Abbreviations

OR: Odds ratio
PAD: Peripheral arterial disease
