# Supplementary material for: Independent factors associated with wearing different types of outdoor footwear in a representative inpatient population: a cross-sectional study
Source: J Foot Ankle Res. 2018 May 29;11:19. doi: 10.1186/s13047-018-0260-7 (PMC5975543; doi:10.1186/s13047-018-0260-7)
Supplement: Supplementary file 1 — Tables S1-S4 include participant characteristics and univariate analyses for each footwear type with a prevalence of > 1% and are available. (DOCX 62 kb) [file 13047_2018_260_MOESM1_ESM.docx]

**Supplementary Tables**

**Supplementary Table 1:** Characteristics and univariate analysis for those participants mostly wearing the outdoor footwear types of running shoes, thongs or walking shoes

| Variables | All | Running shoe | | | Thongs/ Flip Flops | | | Walking Shoe | | |
| --- | --- | --- | --- | --- | --- | --- | --- | --- | --- | --- |
|  |  | n (%) | Odds ratio [95% CI] | *p* Value | n (%) | Odds ratio [95% CI] | *p* Value | n (%) | Odds ratio [95% CI] | *p* Value |
| **Participants^** | 733 | 148 (20.4%) |  |  | 103 (14.2%) |  |  | 98 (13.5%) |  |  |
| **Socio-demographics** |  |  |  |  |  |  |  |  |  |  |
| Age (SD) years | 62.0(18.6) | 62.1(16.7) | 1.00 [0.99-1.01] | 0.897 | 48.3(17.6) | 0.96 [0.95-0.97] | <0.001** | 68.0(18.8) | 1.02 [1.01-1.04] | 0.001** |
| Male sex | 408 (55.8%) | 108 (73.3%) | 2.65 [1.77-3.95] | <0.001** | 46 (45.1%) | 0.61 [0.40-0.93] | 0.021** | 52 (53.1%) | 0.89 [0.58-1.36] | 0.577 |
| Indigenous | 34 (4.6%) | 8 (5.4%) | 1.21 [0.54-2.73] | 0.645 | 7 (6.8%) | 1.61 [0.68-3.79] | 0.279 | 7 (7.1%) | 1.71 [0.72-4.04] | 0.222 |
| Born overseas | 161 (22.0%) | 33 (22.3%) | 1.00 [0.65-1.55] | 0.984 | 25 (24.3%) | 1.14 [0.70-1.86] | 0.592 | 21 (21.6%) | 0.96 [0.57-1.61] | 0.881 |
| <Year 10 Education Level | 395 (54.0%) | 71 (48.0%) | 0.74 [0.52-1.07] | 0.107* | 56 (54.9%) | 1.05 [0.69-1.60] | 0.821 | 60 (61.2%) | 1.42 [0.92-2.19] | 0.117* |
| Socioeconomic Status | 711 |  |  | 0.790 |  |  | 0.037** |  |  | 0.834 |
| Most disadvantaged | 102 (14.4%) | 20 (13.7%) | 1.00 |  | 22 (22.0%) | 1.00 |  | 13 (13.7%) | 1.00 |  |
| Second most disadvantaged | 159 (22.4%) | 32 (21.9%) | 1.04 [0.56-1.94] | 0.910 | 26 (26.0^) | 0.71 [0.38-1.34] | 0.294 | 23 (24.2%) | 1.16 [0.56-2.41] | 0.688 |
| Middle | 98 (13.8%) | 16 (11.5%) | 0.80 [0.39-1.65] | 0.547 | 16 (16.0%) | 0.71 [0.35-1.45] | 0.346 | 16 (16.8%) | 1.34 [0.61-2.95] | 0.472 |
| Second least disadvantaged | 240 (33.8%) | 54 (37.0%) | 1.19 [0.67-2.11] | 0.556 | 23 (23.0%) | 0.38 [0.20-0.27] | 0.003 | 30 (31.6%) | 0.98 [0.49-1.96] | 0.946 |
| Least disadvantaged | 112 (15.8%) | 24 (16.4%) | 1.11 [0.57-2.15] | 0.770 | 13 (13.0%) | 0.47 [0.22-0.99] | 0.048 | 13 (13.7%) | 0.89 [0.39-2.02] | 0.778 |
| Geographic Remoteness | 711 |  |  | 0.764 |  |  | 0.065* |  |  | 0.282 |
| Major city | 435 (61.2%) | 94 (64.4$) | 1.00 |  | 50 (50.0%) | 1.00 |  | 51 (53.7%) | 1.00 |  |
| Inner regional area | 153 (21.5%) | 28 (19.2%) | 0.81 [0.51-1.29] | 0.317 | 23 (23.0%) | 1.36 [0.80-2.31] | 0.264 | 28 (29.5%) | 1.68 [1.01-2.78] | 0.044 |
| Outer regional area | 66 (9.3%) | 14 (9.6%) | 0.96 [0.51-1.81] | 0.905 | 16 (16.0%) | 2.43 [1.29-4.59] | 0.006 | 10 (10.5%) | 1.33 [0.64-2.76] | 0.450 |
| Remote area | 30 (4.2%) | 4 (2.7%) | 0.55 [0.19-1.62] | 0.277 | 6 (6.0%) | 1.90 [0.74-4.87] | 0.182 | 4 (4.2%) | 1.14 [0.38-3.41] | 0.810 |
| Very remote area | 27 (3.8%) | 6 (4.1%) | 1.02 [0.41-2.60] | 0.965 | 5 (5.0%) | 1.73 [0.63-4.77] | 0.291 | 2 (2.1%) | 0.60 [0.14-2.59] | 0.488 |
| **Medical condition history** |  |  |  |  |  |  |  |  |  |  |
| Diabetes | 172 (23.5%) | 28 (18.9%) | 0.71 [0.45-1.12] | 0.138* | 21 (20.4%) | 0.81 [0.48-1.35] | 0.414 | 28 (28.6%) | 1.36 [0.84-2.18] | 0.209 |
| Hypertension | 359 (49.0%) | 63 (42.6%) | 0.73 [0.51-1.05] | 0.092* | 38 (36.9%) | 0.57 [0.37-0.87] | 0.101* | 54 (55.1%) | 1.34 [0.88-2.06] | 0.178* |
| Dyslipidaemia | 234 (31.9%) | 46 (31.1%) | 0.94 [0.64-1.39] | 0.767 | 31 (30.1%) | 0.90 [0.57-1.41] | 0.639 | 36 (36.7%) | 1.27 [0.82-1.98] | 0.291 |
| Myocardial Infarct | 146 (19.9%) | 26 (17.6%) | 0.82 [0.51-1.31] | 0.413 | 16 (15.5%) | 0.70 [0.40-1.24] | 0.226 | 24 (24.5%) | 1.36 [0.82-2.24] | 0.230 |
| Cerebrovascular Accident | 85 (11.6%) | 16 (10.8%) | 0.89 [0.50-1.59] | 0.704 | 6 (5.8%) | 0.43 [0.18-1.00] | 0.051* | 18 (18.4%) | 1.88 [1.07-3.330 | 0.030** |
| Chronic Kidney Disease | 89 (12.1%) | 17 (11.5%) | 0.93 [0.53-1.63] | 0.791 | 7 (6.8%) | 0.49 [0.22-1.09] | 0.079* | 12 (12.2%) | 1.01 [0.53-1.94] | 0.968 |
| Cancer | 174 (23.7%) | 42 (28.4%) | 1.38 [0.92-2.07] | 0.122* | 23 (22.3%) | 0.92 [0.56-1.52] | 0.752 | 23 (23.5%) | 1.00 [0.60-1.64] | 0.983 |
| Arthritis | 274 (37.4%) | 48 (32.4%) | 0.77 [0.53-1.13] | 0.180* | 29 (28.2%) | 0.62 [0.39-0.98] | 0.042** | 53 (54.1%) | 2.23 [1.45-3.43] | <0.001** |
| Depression | 191 (26.1%) | 27 (18.2%) | 0.57 [0.36-0.90] | 0.016** | 36 (35.0%) | 1.65 [1.05-2.57] | 0.027** | 33 (33.7%) | 1.54 [0.97-2.42] | 0.065* |
| Smoker | 104 (14.2%) | 21 (14.2%) | 0.99 [0.59-1.65] | 0.958 | 25 (25.2%) | 2.36 [1.43-3.91] | 0.001** | 11 (11.2%) | 0.73 [0.37-1.41] | 0.348 |
| Ex-Smoker | 304 (41.5%) | 71 (48.0%) | 1.41 [0.98-2.02] | 0.066* | 41 (39.8%) | 0.93 [0.61-1.42] | 0.736 | 36 (36.7%) | 0.80 [0.52-1.24] | 0.322 |
| Mobility impairment | 242 (33.2%) | 44 (29.9%) | 0.84 [0.57-1.25] | 0.388 | 15 (14.7%) | 0.31 [0.17-0.55] | <0.001** | 49 (50.0%) | 2.31 [1.50-3.55] | <0.001** |
| Vision impairment | 110 (15.1%) | 22 (15.1%) | 0.99 [0.60-1.64] | 0.962 | 11 (10.7%) | 0.63 [0.33-1.22] | 0.171* | 17 (17.3%) | 1.20 [0.68-2.12] | 0.523 |
| **Past foot treatment** |  |  |  |  |  |  |  |  |  |  |
| Yes | 256 (34.9%) | 43 (29.1%) | 0.72 [0.49-1.07] | 0.106* | 21 (20.4%) | 0.44 [0.26-0.72] | 0.001** | 46 (46.9%) | 1.81 [1.18-2.79] | 0.007** |
| Podiatry | 180 (24.6%) | 25 (16.9%) | 0.57 [0.35-0.90] | 0.017** | 8 (7.8%) | 0.22 [0.11-0.47] | <0.001** | 37 (37.8%) | 2.10 [1.34-3.28] | 0.001** |
| GP | 93 (12.7%) | 22 (14.9%) | 1.29 [0.77-2.16] | 0.338 | 14 (13.6%) | 1.12 [0.61-2.06] | 0.726 | 11 (11.2%) | 0.87 [0.44-1.69] | 0.674 |
| Surgeon | 36 (4.9%) | 9 (6.1%) | 1.38 [0.63-3.00] | 0.424 | 3 (2.9%) | 0.55 [0.17-1.84] | 0.336 | 5 (5.1%) | 10.7 [0.41-2.83] | 0.889 |
| Specialist Physician | 21 (2.9%) | 5 (3.4%) | 1.23 [0.44-3.41] | 0.693 | 2 (1.9%) | 0.63 [0.14-2.74] | 0.538 | 5 (5.1%) | 2.06 [0.74-5.75] | 0.169* |
| Nurse | 20 (2.7%) | 2 (1.4%) | 0.45 [0.10-1.98] | 0.292 | 1 (1.0%) | 0.33 [0.04-2.50] | 0.283 | 3 (3.1%) | 1.21 [0.35-4.22] | 0.767 |
| Orthotist | 4 (0.5%) | 1 (0.7%) | 1.30 [0.14-12.63] | 0.819 | 1 (1.0%) | 2.03 [0.21-19.67] | 0.543 | 0 | 0 | NA |
| Other | 9 (1.2%) | 1 (0.7%) | 0.49 [0.06-3.91] | 0.496 | 1 (1.0%) | 0.75 [0.09-6.09] | 0.791 | 1 (1.0%) | 0.80 [0.10-6.46] | 0.833 |
| **Foot-related conditions** |  |  |  |  |  |  |  |  |  |  |
| Amputation history | 37 (5.0%) | 9 (6.1%) | 1.43 [0.65-3.14] | 0.369 | 1 (1.0%) | 0.18 [0.02-1.30] | 0.088* | 6 (6.1%) | 1.40 [0.56-3.47] | 0.470 |
| Foot ulcer history | 88 (12.0%) | 16 (10.8%) | 0.86 [0.49-1.54] | 0.618 | 7 (6.8%) | 0.49 [0.22-1.10] | 0.085* | 15 (15.3%) | 1.39 [0.76-2.54] | 0.281 |
| Peripheral neuropathy | 160 (22.0%) | 33 (22.3%) | 1.03 [0.66-1.58] | 0.912 | 11 (10.8%) | 0.39 [0.20-.027] | 0.004** | 29 (29.6%) | 1.60 [1.00-2.58] | 0.051* |
| Foot deformity | 158 (22.4%) | 32 (22.7%) | 1.02 [0.66-1.59] | 0.916 | 8 (8.0%) | 0.26 [0.13-0.56] | <0.001** | 31 (32.0%) | 1.79 [1.12-2.86] | 0.016** |
| PAD severity |  |  |  | 0.463 |  |  | 0.007** |  |  | 0.369 |
| Nil PAD | 575 (79.0%) | 111 (75.0%) | 1.00 |  | 95 (93.1%) | 1.00 |  | 72 (73.5%) | 1.00 |  |
| Mild PAD | 69 (9.5%) | 19 (12.8%) | 1.58 [0.90-2.78] | 0.115 | 2 (2.0%) | 0.15 [0.04-0.62] | 0.009 | 14 (14.3%) | 1.77 [0.94-3.34] | 0.079 |
| Moderate PAD | 51 (7.0%) | 11 (7.4%) | 1.17 [0.58-2.36] | 0.658 | 2 (2.0%) | 0.21 [0.05-0.88] | 0.032 | 7 (7.1%) | 1.13 [0.49-2.61] | 0.774 |
| Critical PAD | 33 (4.5%) | 7 (4.7%) | 1.12 [0.47-2.64] | 0.799 | 3 (2.9%) | 0.50 [0.15-1.68] | 0.263 | 5 (5.1%) | 1.24 [0.46-3.31] | 0.668 |

**p* < 0.2; ***p* < 0.05; ^ 95% CI are for prevalence figure; GP: General Practitioner;

PAD: Peripheral Arterial Disease; SD: standard deviation

**Supplementary Table 2:** Characteristics and univariate analysis for participants mostly wearing the outdoor footwear types of sandals, boots or oxford shoes

| Variables | All | Sandal | | | Boot | | | Oxford shoe | | |
| --- | --- | --- | --- | --- | --- | --- | --- | --- | --- | --- |
|  |  | n (%) | Odds ratio [95% CI] | *p* Value | n (%) | Odds ratio [95% CI] | *p* Value | n (%) | Odds ratio [95% CI] | *p* Value |
| **Participants^** | 733 | 95 (13.1%) |  |  | 78 (10.7%) |  |  | 50 (6.9%) |  |  |
| **Socio-demographics** |  |  |  |  |  |  |  |  |  |  |
| Age (SD) years | 62.0(18.6) | 65.9(17.4) | 1.01 [1.00-1.03] | 0.028** | 53.1(16.1) | 0.97 [0.96-0.99] | <0.001** | 67.4(18.6) | 1.02 [1.00-1.04] | 0.036** |
| Male sex | 408 (55.8%) | 27 (28.4%) | 0.27 [0.17-0.43] | <0.001** | 71 (91.0%) | 9.59 [4.35-21.17] | <0.001** | 44 (88.0%) | 6.44 [2.71-15.30] | <0.001** |
| Indigenous | 34 (4.6%) | 4 (4.2%) | 0.88 [0.30-2.55] | 0.813 | 5 (6.4%) | 1.46 [0.55-3.89] | 0.449 | 1 (2.0%) | 0.40 [0.05-2.97] | 0.368 |
| Born overseas | 161 (22.0%) | 17 (17.9%) | 0.73 [0.42-1.28] | 0.276 | 16 (20.8%) | 0.91 p0.51-1.62] | 0.745 | 18 (36.0%) | 2.09 [1.14-3.83] | 0.017** |
| <Year 10 Education Level | 395 (54.0%) | 53 (56.4%) | 1.12 [0.73-1.74] | 0.600 | 39 (50.0%) | 0.84 [0.53-1.35] | 0.469 | 28 (56.0%) | 1.10 [0.62-1.96] | 0.754 |
| Socioeconomic Status | 711 |  |  | 0.959 |  |  | 0.775 |  |  | 0.150* |
| Most disadvantaged | 102 (14.4%) | 13 (14.3%) | 1.00 |  | 14 (17.9%0 | 1.00 |  | 3 (6.1%) | 1.00 |  |
| Second most disadvantaged | 159 (22.4%) | 23 (25.3%) | 1.16 [0.56-2.41] | 0.688 | 19 (24.4%) | 0.86 [0.41-1.79] | 0.680 | 7 (14.3%) | 1.52 [0.39-6.04] | 0.548 |
| Middle | 98 (13.8%) | 12 (13.2%) | 0.96 [0.41-2.21] | 0.916 | 11 (14.1%) | 0.80 [0.34-1.85] | 0.594 | 7 (14.3%) | 2.54 [0.64-10.12] | 0.186 |
| Second least disadvantaged | 240 (33.8%) | 30 (33.0%) | 0.98 [0.48-1.96] | 0.946 | 22 (28.2%) | 0.63 [0.31-1.29] | 0.210 | 20 (40.8%) | 3.00 [0.87-10.32] | 0.082 |
| Least disadvantaged | 112 (15.8%) | 13 (12.9%) | 0,89 [0.39-2.02] | 0.778 | 12 (15.4%) | 0.75 [0.33-1.70] | 0.485 | 12 (24.5%) | 3.92 [1.07-14.32] | 0.039 |
| Geographic Remoteness | 711 |  |  | 0.356 |  |  | 0.004** |  |  | 0.052* |
| Major city | 435 (61.2%) | 53 (58.2%) | 1.00 |  | 32 (41.0%) | 1.00 |  | 40 (81.6%) | 1.00 |  |
| Inner regional area | 153 (21.5%) | 26 (28.6%) | 1.47 [0.88-2.45] | 0.141 | 24 (30.8%) | 2.33 [1.33-4.11] | 0.003 | 3 (6.1%) | 0.20 [0.06-0.64] | 0.007 |
| Outer regional area | 66 (9.3%) | 5 (5.5%) | 0.58 [0.22-1.52] | 0.269 | 10 (12.8%) | 2.22 [1.04-4.77] | 0.040 | 2 (4.1%) | 0.31 [0.07-1.29] | 0.107 |
| Remote area | 30 (4.2%) | 3 (3.3%) | 0.79 [0.23-2.70] | 0.707 | 7 (9.0%) | 3.79 [1.51-9.49] | 0.005 | 2 (4.1%) | 0.70 [0.16-3.03] | 0.630 |
| Very remote area | 27 (3.8%) | 4 (4.4%) | 1.24 [0.41-3.72] | 0.705 | 5 (6.4%) | 2.83 [1.00-7.96] | 0.049 | 2 (4.1%) | 0.78 [0.18-3.42 | 0.742 |
| **Medical condition history** |  |  |  |  |  |  |  |  |  |  |
| Diabetes | 172 (23.5%) | 28 (29.5%) | 1.43 [0.88-2.30] | 0.146* | 14 (17.9%) | 0.68 [0.37-1.25] | 0.219 | 11 (22.0%) | 0.91 [0.46-1.82] | 0.789 |
| Hypertension | 359 (49.0%) | 53 (55.8%) | 1.38 [0.90-2.14] | 0.143* | 32 (41.0%) | 0.70 [0.44-1.13] | 0.150* | 25 (50.0%) | 1.06 [0.59-1.87] | 0.856 |
| Dyslipidaemia | 234 (31.9%) | 29 (30.5%) | 0.92 [0.58-1.47] | 0.726 | 19 (24.4%) | 0.65 [0.38-1.12] | 0.124* | 18 (36.0%) | 1.21 [0.66-2.20] | 0.540 |
| Myocardial Infarct | 146 (19.9%) | 18 (18.9%) | 0.93 [0.54-1.61] | 0.789 | 10 (12.8%) | 0.56 [0.28-1.12] | 0.099* | 13 (26.0%) | 1.45 [0.75-2.80] | 0.272 |
| Cerebrovascular Accident | 85 (11.6%) | 9 (9.5%) | 0.76 [0.37-1.58] | 0.469 | 7 (9.0%) | 0.72 [0.32-1.62] | 0.429 | 6 (12.0%) | 1.03 [0.43-2.50] | 0.947 |
| Chronic Kidney Disease | 89 (12.1%) | 12 (12.6%) | 10.6 [0.55-2.03] | 0.870 | 6 (7.7%) | 0.58 [0.24-1.37] | 0.210 | 6 (12.0%) | 0.99 [0.41-2.39] | 0.978 |
| Cancer | 174 (23.7%) | 21 (22.1%) | 0.91 [0.54-1.53] | 0.721 | 12 (15.4%) | 0.56 [0.30-1.06] | 0.075* | 13 (26.0%) | 1.15 [0.60-2.22] | 0.673 |
| Arthritis | 274 (37.4%) | 36 (37.9%) | 10.4 [0.66-1.62] | 0.879 | 23 (29.5%) | 0.68 [0.41-1.13] | 0.138* | 16 (32.0%) | 0.78 [0.42-1.45] | 0.432 |
| Depression | 191 (26.1%) | 27 (28.4%) | 1.15 [0.71-1.86] | 0.570 | 17 (21.8%) | 0.77 [0.44-1.36] | 0.368 | 9 (18.0%) | 0.61 [0.29-1.27] | 0.184* |
| Smoker | 104 (14.2%) | 1 (1.1%) | 0.056 [0.01-0.40] | 0.004** | 23 (29.5%) | 2.93 [1.71-5.02] | <0.001** | 4 (8.0%) | 0.50 [0.18-1.42] | 0.194* |
| Ex-Smoker | 304 (41.5%) | 47 (49.5%) | 1.46 [0.95-2.26] | 0.085 | 29 (37.2%) | 0.82 [0.51-1.34] | 0.432 | 24 (48.0%) | 1.24 [0.75-2.38] | 0.322 |
| Mobility impairment | 242 (33.2%) | 40 (42.1%) | 1.58 [1.02-2.45] | 0.042** | 9 (11.7%) | 0.24 [0.12-0.490 | <0.001** | 14 (28.0%) | 0.78 [0.41-1.48] | 0.444 |
| Vision impairment | 110 (15.1%) | 18 (18.9%) | 1.36 [0.78-2.39] | 0.276 | 7 (9.0%) | 0.52 [0.23-1.16] | 0.111* | 6 (12.0%) | 0.75 [0.31-1.80] | 0.520 |
| **Past Foot Treatment** |  |  |  |  |  |  |  |  |  |  |
| Yes | 256 (34.9%) | 40 (42.1%) | 1.44 [0.93-2.23] | 0.106* | 17 (21.8%) | 0.49 [0.28-0.86] | 0.013** | 15 (30.0%) | 0.79 [0.43-1.48] | 0.469 |
| Podiatry | 180 (24.6%) | 30 (31.6%) | 1.51 [0.94-2.41] | 0.088* | 5 (6.4%) | 0.19 [0.08-0.47] | <0.001** | 12 (24.0%) | 0.97 [0.50-1.90] | 0.930 |
| GP | 93 (12.7%) | 13 (13.7%) | 1.12 [0.60-2.11] | 0.717 | 9 (11.5%) | 0.90 [0.43-1.87] | 0.779 | 4 (8.0%) | 0.59 [0.21-1.68] | 0.321 |
| Surgeon | 36 (4.9%) | 6 (6.3%) | 1.40 [0.57-3.47] | 0.468 | 3 (3.8%) | 0.77 [0.23-2.58] | 0.671 | 0 | 0 | NA |
| Specialist Physician | 21 (2.9%) | 3 (3.2%) | 1.11 [0.32-3.84] | 0.869 | 1 (1.3%) | 0.41 [0.05-3.08] | 0.385 | 0 | 0 | NA |
| Nurse | 20 (2.7%) | 5 (5.3%) | 4.45 [0.86-6.96] | 0.093* | 1 (1.3%) | 0.46 [0.06-3.45] | 0.446 | 0 | 0 | NA |
| Orthotist | 4 (0.5%) | 0 | 0 | NA | 0 | 0 | NA | 0 | 0 | NA |
| Other | 9 (1.2%) | 3 (3.2%) | 3.40 [0.84-13.82] | 0.088* | 1 | 1.04 [0.13-8.42] | 0.971 | 1 (2.0%) | 1.70 [0.21-13.90] | 0.619 |
| **Foot-related conditions** |  |  |  |  |  |  |  |  |  |  |
| Amputation history | 37 (5.0%) | 3 (3.2%) | 0.63 [0.19-2.11] | 0.454 | 1 (1.3%) | 0.24 [0.03-1.80] | 0.165* | 4 (8.0%) | 1.87 [0.63-5.54] | 0.257 |
| Foot ulcer history | 88 (12.0%) | 17 (17.9%) | 1.74 [0.98-3.12] | 0.061* | 4 (5.2%) | 0.37 [0.13-1.05] | 0.061* | 5 (10.0%) | 0.80 [0.31-2.08] | 0.653 |
| Peripheral neuropathy | 160 (22.0%) | 21 (22.3%) | 1.03 [0.61-1.73] | 0.924 | 9 (11.5%) | 0.43 [0.21-0.89] | 0.022** | 14 (28.0%) | 1.42 [0.75-2.70] | 0.287 |
| Foot deformity | 158 (22.4%) | 26 (27.7%) | 1.39 [0.85-2.28] | 0.187* | 5 (6.7%) | 0.22 [0.09-0.56] | 0.001** | 11 (23.4%) | 1.07 [0.53-2.15] | 0.859 |
| PAD severity |  |  |  | 0.486 |  |  | 0.132* |  |  | 0.532 |
| Nil PAD | 575 (79.0%) | 72 (76.6%) | 1.00 |  | 69 (88.5%) | 1.00 |  | 36 (72.0%) | 1.00 |  |
| Mild PAD | 69 (9.5%) | 7 (7.4%) | 0.78 [0.35-1.78] | 0.561 | 7 (9.0%) | 0.82 [0.36-1.87] | 0.642 | 7 (14.0%) | 1.68 [0.72-3.94] | 0.232 |
| Moderate PAD | 51 (7.0%) | 9 (9.6%) | 1.52 [0.71-3.27] | 0.279 | 1 (1.3%) | 0.15 [0.02-1.10] | 0.061 | 5 (10.0%) | 1.65 [0.62-4.42] | 0.316 |
| Critical PAD | 33 (4.5%) | 6 (6.4%) | 1.54 [0.62-3.87] | 0.355 | 1 (1.3%) | 0.23 [0.03-1.69] | 0.148 | 2 (4.0%) | 0.96 [0.22-4.17] | 0.957 |

**p* < 0.2; ***p* < 0.05; ^ 95% CI are for prevalence figure; GP: General Practitioner;

PAD: Peripheral Arterial Disease; SD: Standard deviation.

**Supplementary Table 3:** Characteristics and univariate analysis for participants mostly wearing the outdoor footwear types of court shoes, moccasins or slippers

| Variables | All | Court shoe | | | Moccasin | | | Slipper | | |
| --- | --- | --- | --- | --- | --- | --- | --- | --- | --- | --- |
|  |  | n (%) | Odds ratio [95% CI] | *p* Value | n (%) | Odds ratio [95% CI] | *p* Value | n (%) | Odds ratio [95% CI] | *p* Value |
| **Participants^** | 733 | 49 (6.7%) |  |  | 42 (5.8%) |  |  | 20 (2.8%) |  |  |
| **Socio-demographics** |  |  |  |  |  |  |  |  |  |  |
| Age (SD) years | 62.0(18.6) | 72.9(14.9) | 1.05 [0.02-1.07] | <0.001** | 65.2(17.3) | 1.01 [0.99-1.03] | 0.242 | 72.3(16.7) | 1.04 [1.01-1.08] | 0.011** |
| Male sex | 408 (55.8%) | 1 (2.0%) | 0.01 [0.00-0.10] | <0.001** | 16 (38.1%) | 0.47 [0.25-0.89] | 0.021** | 18 (90.0%) | 7.46 [1.72-32.38] | 0.007** |
| Indigenous | 34 (4.6%) | 0 | 0 | NA | 0 | 0 | NA | 0 | 0 | NA |
| Born overseas | 161 (22.0%) | 9 (18.4%) | 0.77 [0.37-1.63] | 0.501 | 4 (9.5%) | 0.35 [0.12-1.00] | 0.050* | 7 (35.0%) | 1.92 [0.75-4.90] | 0.171* |
| <Year 10 Education Level | 395 (54.0%) | 27 (55.1%) | 1.06 [0.59-1.89] | 0.858 | 24 (57.1%) | 1.15 [0.61-2.16] | 0.661 | 13 (65.0%) | 1.61 [0.64-4.09] | 0.315 |
| Socioeconomic Status | 711 |  |  | 0.277 |  |  | 0.678 |  |  | 0.785 |
| Most disadvantaged | 102 (14.4%) | 4 (8.9%) | 1.00 |  | 6 (14.6%) | 1.00 |  | 1 (5.0%) | 1.00 |  |
| Second most disadvantaged | 159 (22.4%) | 6 (13.3%) | 0.96 [0.27-3.50] | 0.955 | 8 (19.5%) | 0.85 [0.29-2.53] | 0.770 | 5 (25.0%) | 3.29 [0.38-28.58] | 0.280 |
| Middle | 98 (13.8%) | 9 (20.0%) | 2.48 [0.74-8.34] | 0.142 | 4 (9.8%) | 0.68 [0.19-2.49] | 0.562 | 2 (10.0% | 2.11 [0.19-23.60] | 0.546 |
| Second least disadvantaged | 240 (33.8%) | 16 (35.6%) | 1.75 [0.57-5.36] | 0.329 | 18 (43.9%) | 1.30 [0.50-3.37] | 0.595 | 11 (55.0%) | 4.85 [0.62-38.04] | 0.133 |
| Least disadvantaged | 112 (15.8%) | 10 (22.2%) | 2.38 [0.72-7.83] | 0.155 | 5 (12.2%) | 0.74 [0.22-2.50] | 0.628 | 1 (5.0%) | 0.90 [0.06-14.59] | 0.941 |
| Geographic Remoteness | 711 |  |  | 0.216 |  |  | 0.904 |  |  | 0.598 |
| Major city | 435 (61.2%) | 36 (80.0%) | 1.00 |  | 29 (70.7%) | 1.00 |  | 15 (75.0%) | 1.00 |  |
| Inner regional area | 153 (21.5%) | 5 (11.1%) | 0.37 [0.14-0.97] | 0.042 | 8 (19.5%) | 0.77 [0.34-1.72] | 0.521 | 2 (10.0%) | 0.37 [0.08-1.63] | 0.189 |
| Outer regional area | 66 (9.3%) | 2 (4.4%) | 0.34 [0.08-1.46] | 0.146 | 3 (7.3%) | 0.66 [0.20-2.23] | 0.501 | 2 (10.0%) | 0.87 [0.19-3.87] | 0.849 |
| Remote area | 30 (4.2%) | 2 (4.4%) | 0.78 [0.18-3.42] | 0.743 | 0 | 0 | NA | 1 (5.0%) | 0.95 [0.12-7.48] | 0.964 |
| Very remote area | 27 (3.8%) | 0 | 0 | NA | 1 (2.4%) | 0.53 [ 0.07-4.06] | 0.543 | 0 | 0 | NA |
| **Medical condition history** |  |  |  |  |  |  |  |  |  |  |
| Diabetes | 172 (23.5%) | 8 (16.3%) | 0.62 [0.28-1.34] | 0.221 | 11 (26.2%) | 1.16 [0.57-2.36] | 0.678 | 6 (30.0%) | 1.41 [0.53-3.72] | 0.493 |
| Hypertension | 359 (49.0%) | 27 (55.1%) | 1.31 [0.73-2.35] | 0.359 | 26 (61.9%) | 1.76 [0.93-3.35] | 0.083* | 12 (60.0%) | 1.60 [0.65-3.95] | 0.312 |
| Dyslipidaemia | 234 (31.9%) | 19 (38.8%) | 1.37 [0.75-2.49] | 0.301 | 16 (38.1%) | 1.32 [0.70-2.52] | 0.392 | 6 (30.0%) | 0.90 [0.34-2.38] | 0.839 |
| Myocardial Infarct | 146 (19.9%) | 12 (24.5%) | 1.33 [0.67-2.61] | 0.414 | 11 (26.2%) | 1.46 [0.71-2.97] | 0.302 | 5 (25.0%) | 1.35 [0.48-3.77] | 0.570 |
| Cerebrovascular Accident | 85 (11.6%) | 7 (14.3%) | 1.28 [0.56-2.95] | 0.562 | 7 (16.7%) | 1.55 [0.67-3.62] | 0.307 | 3 (15.0%) | 1.34 [0.39-4.68] | 0.644 |
| Chronic Kidney Disease | 89 (12.1%) | 4 (8.2%) | 0.63 [0.22-1.79] | 0.383 | 7 (16.7%) | 1.49 [0.64-3.46] | 0.355 | 8 (40.0%) | 5.22 [2.07-13.15] | <0.001** |
| Cancer | 174 (23.7%) | 13 (26.5%) | 1.19 [0.61-2.29] | 0.611 | 9 (21.4%) | 0.88 [0.41-1.88] | 0.738 | 3 (15.0%) | 0.57 [0.16-1.95] | 0.367 |
| Arthritis | 274 (37.4%) | 25 (51.0%) | 1.84 [1.03-3.29] | 0.040** | 18 (42.9%) | 1.29 [0.68-2.42] | 0.435 | 5 (25.0%) | 0.56 [0.20-1.54] | 0.259 |
| Depression | 191 (26.1%) | 17 (34.7%) | 1.56 [0.85-2.88] | 0.155* | 9 (21.4%) | 0.76 [0.36-1.63] | 0.485 | 4 (20.0%) | 0.70 [0.23-2.13] | 0.535 |
| Smoker | 104 (14.2%) | 3 (6.1%) | 0.37 [0.11-1.22] | 0.102* | 3 (7.1%) | 0.44 [0.14-1.46] | 0.182* | 2 (10.0%) | 0.66 [0.15-2.88] | 0.578 |
| Ex-Smoker | 304 (41.5%) | 13 (26.5%) | 0.49 [0.26-0.94] | 0.032** | 15 (35.7%) | 0.78 [0.41-1.49] | 0.448 | 11 (55.0%) | 1.76 [0.72-4.31] | 0.213 |
| Mobility impairment | 242 (33.2%) | 23 (46.9%) | 1.89 [1.05-3.39] | 0.033** | 16 (38.1%) | 1.27 [0.67-2.42] | 0.463 | 8 (40.0%) | 1.37 [0.55-3.40] | 0.496 |
| Vision impairment | 110 (15.1%) | 5 (10.2%) | 0.62 [0.14-1.59] | 0.318 | 8 (19.0%) | 1.34 [0.60-2.97] | 0.475 | 6 (30.0%) | 2.47 [0.93-6.58] | 0.070* |
| **Past foot treatment** |  |  |  |  |  |  |  |  |  |  |
| Yes | 256 (34.9%) | 26 (53.1%) | 2.26 [1.26-4.04] | 0.006** | 14 (33.3%) | 0.94 [0.48-1.81] | 0.847 | 6 (30.0%) | 0.80 [0.30-2.11] | 0.654 |
| Podiatry | 180 (24.6%) | 24 (49.0%) | 3.26 [1.18-5.87] | <0.001** | 12 (28.6%) | 1.25 [0.63-2.49] | 0.530 | 6 (30.0%) | 1.33 [0.50-3.52] | 0.565 |
| GP | 93 (12.7%) | 6 (12.2%) | 0.97 [0.40-2.35] | 0.949 | 3 (7.1%) | 0.52 [0.16-1.72] | 0.285 | 1 (5.0%) | 0.36 [ 0.05-2.72] | 0.323 |
| Surgeon | 36 (4.9%) | 1 (2.0%) | 0.39 [0.05-2.94] | 0.364 | 2 (4.8%) | 0.99 [0.23-4.26] | 0.985 | 1 (5.0%) | 1.04 [0.14-8.00] | 0.970 |
| Specialist Physician | 21 (2.9%) | 1 (2.0%) | 0.68 [0.09-5.21] | 0.714 | 1 (2.4%) | 0.81 [0.11-6.18] | 0.839 | 0 | 0 | NA |
| Nurse | 20 (2.7%) | 1 (2.0%) | 0.76 [0.10-5.84] | 0.794 | 1 (2.4%) | 0.90 [0.12-6.93] | 0.921 | 0 | 0 | NA |
| Orthotist | 4 (0.5%) | 0 | 0 | NA | 1 (2.4%) | 5.54 [0.56-54.40] | 0.142* | 0 | 0 | NA |
| Other | 9 (1.2%) | 0 | 0 | NA | 0 | 0 | NA | 0 | 0 | NA |
| **Foot-related conditions** |  |  |  |  |  |  |  |  |  |  |
| Amputation history | 37 (5.0%) | 2 (4.1%) | 0.86 [0.20-3.69] | 0.837 | 2 (4.8%) | 1.02 [0.24-4.40] | 0.980 | 0 | 0 | NA |
| Foot ulcer history | 88 (12.0%) | 3 (6.1%) | 0.46 [0.14-1.51] | 0.200 | 5 (11.9%) | 0.99 [0.38-2.59] | 0.984 | 2 (10.0%) | 0.81 [0.19-3.55] | 0.781 |
| Peripheral neuropathy | 160 (22.0%) | 11 (22.4%) | 10.3 [0.51-2.07] | 0.932 | 5 (11.9%) | 0.46 [0.18-1.20] | 0.113* | 6 (30.0%) | 1.54 [0.58-4.08] | 0.382 |
| Foot deformity | 158 (22.4%) | 13 (28.3%) | 1.40 [0.72-2.73] | 0.323 | 10 (24.4%) | 1.13 [0.54-2.36] | 0.749 | 7 (35.0%) | 1.91 [0.75-4.87] | 0.176* |
| PAD severity |  |  |  | 0.660 |  |  | 0.644 |  |  | 0.079* |
| Nil PAD | 575 (79.0%) | 39 (27.6%) | 1.00 |  | 34 (81.0%) | 1.00 |  | 12 (60.0%) | 1.00 |  |
| Mild PAD | 69 (9.5%) | 4 (8.2%) | 0.84 [0.29-2.43] | 0.749 | 2 (4.8%) | 0.47 [0.11-2.01] | 0.310 | 2 (10.0%) | 1.39 [0.31-6.36] | 0.669 |
| Moderate PAD | 51 (7.0%) | 5 (10.2%) | 1.52 [0.57-4.04] | 0.403 | 3 (7.1%) | 1.01 [0.30-3.41] | 0.987 | 3 915.0%) | 3.00 [0.81-10.93] | 0.100 |
| Critical PAD | 33 (4.5%) | 1 (2.0%) | 0.43 [0.06-3.21] | 0.408 | 3 (7.1%) | 1.58 [0.46-5.45] | 0.467 | 3 (15.0%) | 4.67 [1.25-17.42] | 0.022 |

**p* < 0.2; ***p* < 0.05; ^ 95% CI are for prevalence figureGP: General Practitioner;

PAD: Peripheral Arterial Disease; SD: Standard deviation.

**Supplementary Table 4:** Characteristics and univariate analysis for participants mostly wearing the outdoor footwear types of bespoke footwear or barefoot (no footwear).

| Variables | All | Bespoke footwear | | | Barefoot | | |
| --- | --- | --- | --- | --- | --- | --- | --- |
|  |  | n (%) | Odds ratio [95% CI] | *p* Value | n (%) | Odds ratio [95% CI] | *p* Value |
| **Participants^** | 733 | 12 (1.7%) |  |  | 12 (1.7%) |  |  |
| **Socio-demographics** |  |  |  |  |  |  |  |
| Age (SD) years | 62.0(18.6) | 65.8(15.1) | 1.01 [0.98-1.05] | 0.480 | 54.3(17.5) | 0.98 [0.95-1.01] | 0.156* |
| Male sex | 408 (55.8%) | 7 (58.3%) | 1.12 [0.35-3.55] | 0.851 | 8 (66.7%) | 1.61 [0.48-5.38] | 0.443 |
| Indigenous | 34 (4.6%) | 0 | 0 | NA | 0 | 0 | NA |
| Born overseas | 161 (22.0%) | 3 (25.0%) | 1.17 [0.31-4.37] | 0.817 | 3 (25.0%) | 1.17 [0.31-4.37] | 0.817 |
| <Year 10 Education Level | 395 (54.0%) | 5 (41.7%) | 0.61 [0.19-1.93] | 0.607 | 5 (41.7%) | 0.61 [0.19-1.93] | 0.397 |
| Socioeconomic Status | 711 |  |  | 0.362 |  |  | 0.744 |
| Most disadvantaged | 102 (14.4%) | 0 | 1.00 |  | 3 (30.0%) | 1.00 |  |
| Second most disadvantaged | 159 (22.4%) | 2 (16.7%) | NA |  | 3 (30.0%) | 0.64 [0.13-3.22] | 0.585 |
| Middle | 98 (13.8%) | 2 (16.7%) | NA |  | 0 | 0 | NA |
| Second least disadvantaged | 240 (33.8%) | 4 (33.3%) | NA |  | 2 (20.0%) | 0.28 [0.05-1.68] | 0.163 |
| Least disadvantaged | 112 (15.8%) | 4 (33.3%) | NA |  | 2 (20.0%) | 0.59 [0.10-3.63] | 0.573 |
| Geographic Remoteness | 711 |  |  | 0.307 |  |  | 0.840 |
| Major city | 435 (61.2%) | 10 (83.3%) | 1.00 |  | 8 (80.0%) | 1.00 |  |
| Inner regional area | 153 (21.5%) | 0 | 0 | NA | 1 (10.0%) | 0.35 [0.04-2.82] | 0.323 |
| Outer regional area | 66 (9.3%) | 1 (8.3%) | 0.64 [0.08-5.13] | 0.680 | 0 | 0 | NA |
| Remote area | 30 (4.2%) | 0 | 0 | NA | 1 (10.0%) | 1.82 [0.22-15.04] | 0.579 |
| Very remote area | 27 (3.8%) | 1 (8.3%) | 1.62 [0.20-12.11] | 0.653 | 0 | 0 | NA |
| **Medical condition history** |  |  |  |  |  |  |  |
| Diabetes | 172 (23.5%) | 6 (50.0%) | 3.33 [1.06-10.46] | 0.040** | 4 (33.3%) | 1.64 [0.49-5.51] | 0.425 |
| Hypertension | 359 (49.0%) | 9 (75.0%) | 3.21 [0.86-11.95] | 0.082* | 6 (50.0%) | 1.05 [0.34-3.29] | 0.931 |
| Dyslipidaemia | 234 (31.9%) | 4 (33.3%) | 1.06 [0.32-3.55] | 0.926 | 4 (33.3%) | 1.05 [0.32-3.55] | 0.926 |
| Myocardial Infarct | 146 (19.9%) | 4 (33.3%) | 2.03 [0.60-6.84] | 0.252 | 2 (16.7%) | 0.80 [0.17-3.69] | 0.773 |
| Cerebrovascular Accident | 85 (11.6%) | 4 (33.3%) | 3.90 [1.15-13.27] | 0.029** | 1 (8.3%) | 0.68 [0.09-5.35] | 0.716 |
| Chronic Kidney Disease | 89 (12.1%) | 4 (33.3%) | 3.75 [1.11-12.72] | 0.034** | 3 (25.0%) | 2.47 [0.66-9.29] | 0.182* |
| Cancer | 174 (23.7%) | 3 (25.0%) | 1.08 [0.29-4.05] | 0.905 | 5 (41.7%) | 2.36 [0.74-7.53] | 0.147* |
| Arthritis | 274 (37.4%) | 5 (41.7%) | 1.21 [0.38-3.85] | 0.747 | 4 (33.3%) | 0.84 [0.25-2.82] | 0.781 |
| Depression | 191 (26.1%) | 3 (25.0%) | 0.95 [0.25-3.53] | 0.934 | 3 (25.0%) | 0.95 [0.25-3.53] | 0.934 |
| Smoker | 104 (14.2%) | 2 (16.7%) | 1.20 [0.26-5.56] | 0.816 | 3 (25.0%) | 2.02 [0.54-7.60] | 0.297 |
| Ex-Smoker | 304 (41.5%) | 6 (50.0%) | 1.43 [0.46-4.47] | 0.540 | 4 (33.3%) | 0.71 [0.21-2.37] | 0.573 |
| Mobility impairment | 242 (33.2%) | 5 (41.7%) | 1.47 [0.46-4.67] | 0.518 | 6 (50.0%) | 2.07 [0.66-6.47] | 0.214 |
| Vision impairment | 110 (15.1%) | 4 (33.3%) | 2.86 [0.85-9.66] | 0.091* | 2 (16.7%) | 1.12 [0.24-5.28] | 0.886 |
| **Past foot treatment** |  |  |  |  |  |  |  |
| Yes | 256 (34.9%) | 9 (75.0%) | 5.82 [1.56-21.68] | 0.009** | 4 (33.3%) | 0.94 [0.28-3.15] | 0.920 |
| Podiatry | 180 (24.6%) | 9 (75.0%) | 9.68 [2.59-36.14] | 0.001** | 1 (8.3%) | 0.28 [0.04-2.15] | 0.219 |
| GP | 93 (12.7%) | 4 (33.3%) | 3.60 [1.06-12.22] | 0.040** | 1 (8.3%) | 0.63 [0.08-4.94] | 0.660 |
| Surgeon | 36 (4.9%) | 4 (33.3%) | 11.02 [3.15-38.58] | <0.001** | 0 | 0 | NA |
| Specialist Physician | 21 (2.9%) | 2 (16.7%) | 7.32 [1.50-35.70] | 0.014** | 1 (8.3%) | 3.16 [0.39-25.63] | 0.282 |
| Nurse | 20 (2.7%) | 3 (25.0%) | 14.54 [3.60-58.82] | <0.001** | 1 (8.3%) | 3.52 [0.43-28.70] | 0.241 |
| Orthotist | 4 (0.5%) | 1 (8.3%) | 21.55 [2.08-223.72] | 0.010** | 0 | 0 | NA |
| Other | 9 (1.2%) | 0 | 0 | NA | 1 (8.3%) | 8.02 [0.92-69.74] | 0.059* |
| **Foot Disease History** |  |  |  |  |  |  |  |
| Amputation history | 37 (5.0%) | 5 (41.7%) | 16.87 [5.05-56.37] | <0.001** | 0 | 0 | NA |
| Foot ulcer history | 88 (12.0%) | 5 (41.7%) | 5.50 [1.71-17.72] | 0.004** | 2 (16.7%) | 1.48 [0.32-6.85] | 0.618 |
| Peripheral neuropathy | 160 (22.0%) | 6 (50.0%) | 3.65 [1.16-11.49] | 0.027** | 7 (58.3%) | 5.16 [1.61-16.48] | 0.006** |
| Foot deformity | 158 (22.4%) | 6 (50.0%) | 3.57 [1.14-11.23] | 0.030** | 3 (25.0%) | 1.16 [0.31-4.34] | 0.825 |
| PAD severity |  |  |  | 0.681 |  |  | 0.414 |
| Nil PAD | 575 (79.0%) | 9 (75.0%) | 1.00 |  | 8 (66.7%) | 1.00 |  |
| Mild PAD | 69 (9.5%) | 1 (8.3%) | 0.92 [0.12-7.37] | 0.937 | 3 (25.0%) | 3.21 [0.83-12.38] | 0.091 |
| Moderate PAD | 51 (7.0%) | 2 (16.7%) | 2.61 [0.55-12.41] | 0.229 | 1 (8.3%) | 1.44 [0.18-11.74] | 0.734 |
| Critical PAD | 33 (4.5%) | 0 | 0 | NA | 0 | 0 | NA |

**p* < 0.2; ***p* < 0.05; ^ 95% CI are for prevalence figure; GP: General Practitioner;

PAD: Peripheral Arterial Disease; SD: Standard deviation.
